# Deep learning predictions on a new dataset: Natural gas production and liquid level detection

**DOI:** 10.1371/journal.pone.0333905

**Published:** 2025-10-09

**Authors:** Dong Wang, Jian Lian, Chengjiang Li, Yanlei Wang

**Affiliations:** 1 School of Information Science and Electrical Engineering, Shandong Jiaotong University, Jinan, Shandong, China; 2 School of Intelligence Engineering, Shandong Management University, Jinan, Shandong, China; 3 Swinburne University of Technology, Shandong University of Sci & Tech, Jinan, Shandong, China; 4 Shandong University of Political Science and Law, Jinan, Shandong, China; University of Oklahoma, UNITED STATES OF AMERICA

## Abstract

In the energy sector, accurate forecasting of natural gas production and liquid level detection is crucial for efficient resource management and operational planning. This study proposes an integrated deep learning model by incorporating bidirectional long short-term memory and Informer, for predicting these critical parameters. The bidirectional long short-term memory model, a type of recurrent neural network, is renowned for its ability to capture temporal dependencies in sequential data, making it a strong candidate for time series forecasting. On the other hand, Informer, a recent advancement in the field, offers an innovative self-attention mechanism that can handle long-term dependencies with reduced computational complexity. In addition, these models are implemented by using a comprehensive dataset of natural gas production and liquid level detection, applying rigorous preprocessing and feature engineering techniques to enhance model performance. The proposed deep learning models are evaluated on the dataset comparing with the state-of-the-art algorithms. Experimental results demonstrate the effectiveness of both models for gas production and liquid level detection, simultaneously. This study contributes to the body of knowledge by providing insights into the application of advanced deep learning techniques in the energy sector and offers a benchmark for future research in this domain.

## Introduction

The production of natural gas is subject to various geological and operational factors that can influence its viscosity and liquid level [1]. The accurate prediction of these parameters is essential for optimizing extraction processes and managing the economic viability of operations. Traditional forecasting methods often rely on empirical models, which may not fully capture the complexity of geological formations and production dynamics [2]. However, with the advent of machine learning, there is a paradigm shift towards data-driven approaches that can offer more precise predictions [3,4]. The integration of machine learning into operational planning (MLOPs) has emerged as a key strategy for deploying software systems in production [5]. In addition, MLOPs can facilitate the collaboration between developers, data scientists, and operators [6]. This is particularly important in the energy industry [7–9]. The application of machine learning techniques for energy industry has been growing, with a focus on optimizing energy management [10,11]. Moreover, a bibliographic review indicates that artificial intelligence (AI) and machine learning have become increasingly essential in energy-related research fields [12].

The significance of natural gas production prediction and liquid level detection [13] cannot be overstated. It lies in the ability to harness vast amounts of data generated throughout the oil and gas industry’s various processes [14]. The application of machine learning is particularly crucial due to the inherent complexity and nonlinearity of the petroleum reservoir behavior [15]. The machine learning models, such as Artificial Neural Networks (ANNs), have demonstrated their promising outcomes in handling such complexities [16]. Moreover, the precision of these predictions is vital for optimizing gas engineering processes [17]. And accurate predictions can significantly enhance operational efficiency and reduce exploration risks [18]. The use of machine learning also extends to real-time monitoring and predictive maintenance, which can preempt equipment failures and reduce downtime [19,20]. The integration of machine learning into energy industry represents a significant leap forward in terms of predictive capabilities [21]. It is a testament to the power of data-driven approaches in addressing the intricate challenges of this sector [22].

The algorithms presented in the natural gas production prediction and liquid level detection can be roughly divided into two categories, including machine learning-based and deep learning-based. On one hand, machine learning has emerged as a critical tool in the oil and gas industry for a long time. A comprehensive review [2] of the field highlights the application of machine learning models in various aspects, including geophysics, geological modeling, reservoir engineering, and production engineering. Recent advancements in machine learning have shown promising outcomes for natural gas production and liquid level detection predictions. For instance, Mukherjee et al. [23] introduced a group of supervised regression methods with high predictive performance compared with state-of-the-art methods for estimating undrilled well production. A machine learning model was conducted using the data from an active gas field. Both the geological, drilling, and production information were leveraged as direct and indirect factors for controlling production. Accordingly, linear regression (LR), principal component analysis, neural network (NN) regression, boosted Decision tree (DT), and binned DT were used to find the optimal prediction of the gas field. To incorporate comprehensive site-specific geological and operational factors, Hui et al. [24] proposed a comprehensive machine learning approach to predict the shale gas production using both the geological and operational information. In their study, four machine learning models (NN, extra trees, gradient boosting DT, and LR) were evaluated. Van Hung et al. [25] proposed an approach using regression algorithms and ensemble methods to leverage the results. A typical Recurrent Neural Network (RNN) using Long Short-Term Memory (LSTM) and Gated Recurrent Unit (GRU), has been developed to predict gas production. The work of [3] tried to model the global annual natural gas production with various types of machine learning algorithms to implement production prediction and obtain a peak production date.

On the other hand, deep learning has been particularly impactful in processing large volumes of data and capturing complex patterns. The application of deep learning in the oil and gas industry shows promise in areas such as seismic data processing and reservoir characterization. For instance, [26] employed a modified LSTM architecture for predicting the gas flow rates in nature gas wells. And the Ensemble Kalman Filter (EnKF) method was exploited to update the flow rate predictions. Shi et al. [27] proposed a hybrid physics guided variational Bayesian spatial-temporal NN. Zha et al. [28] proposes a Convolutional Neural Network (CNN)-LSTM model to predict gas field production for a gas field in China. By combining CNN and LSTM, the presented model predicted the varied trends of gas field production. Ma et al. [29] introduced a hybrid NN model to predict shale gas production. This model can capture sequential dependencies within production data and the nonlinear correlations between production and governing factors. The comparative analysis demonstrated the superior performance of this model over the LSTM and GRU models in both short-term and long-term prediction. In the work of [30], a deep learning model integrated with a decline curve analysis model and production data was proposed for gas well production prediction, leveraging LSTM due to the time-series structure of the data.

In general, past studies have inadequately integrated the capture of both short-term temporal fluctuations and long-term dependencies in natural gas production and liquid level data, with few models combining bidirectional sequence learning and efficient long-sequence feature extraction for these tasks. Specifically, forecasting natural gas production involves complex, nonlinear interactions between geological factors and operational variables, making it challenging for traditional models to balance short-term fluctuations and long-term trends. For liquid level detection, distinguishing between severe, moderate, and no accumulation requires identifying subtle time-series patterns often masked by noise or operational interruptions—an area where conventional machine learning and even some deep learning models struggle, as they lack mechanisms to prioritize critical temporal features.

Bearing the above-mentioned analysis in mind, this study proposes a novel model that combines Bidirectional Long Short-Term Memory (Bi-LSTM) [31] and Informer [32]. The Bi-LSTM is leveraged for processing sequential data. It can learn both forward and backward temporal dependencies. This is essential as the trends in factors such as pressure, temperature, and flow rate can have significant impacts on natural gas production and liquid accumulation. The Informer serves as the back-end. It employs a probabilistic attention mechanism that not only reduces computational complexity and memory requirements compared to standard transformer models but also enables the model to focus on the most relevant parts of the input data. Additionally, the distillation process in the Informer highlights the most salient features, generating a more concentrated and informative feature map for subsequent analysis. This combination of Bi-LSTM and Informer allows the proposed model to handle long-sequence data more efficiently, extract hierarchical and relevant features, and make more accurate predictions and classifications. It represents a significant improvement over traditional models in the context of natural gas production and wellhead liquid accumulation analysis, providing a more powerful and adaptable tool for the energy industry.

In general, the contributions of this study include the followings:

- This study proposes a hybrid model of Bi-LSTM and Informer. The Bi-LSTM adeptly handles sequential data by learning bidirectional temporal relationships. The Informer can optimize feature extraction and reduce computational load.
- This study devises an effective data processing pipeline. It tackles data scarcity and noise by employing advanced preprocessing and augmentation methods.
- The experimental results demonstrate the superiority of the proposed approach over the state-of-the-art techniques.

## Methodology

The proposed model combines the strengths of Bi-LSTM and Informer to address the challenges of natural gas production prediction and liquid level detection in natural gas wellheads. The model is structured in a sequential manner, with Bi-LSTM serving as the front-end and Informer as the back-end.

### Bi-LSTM

The proposed model uses the Bi-LSTM model [33] as the core framework. Ever since the initial LSTM model was introduced by Hochreiter and Schmidhuber [34], its variant models have been extensively utilized in natural language processing (NLP) [35] tasks with favorable results.

**Fig 1:**
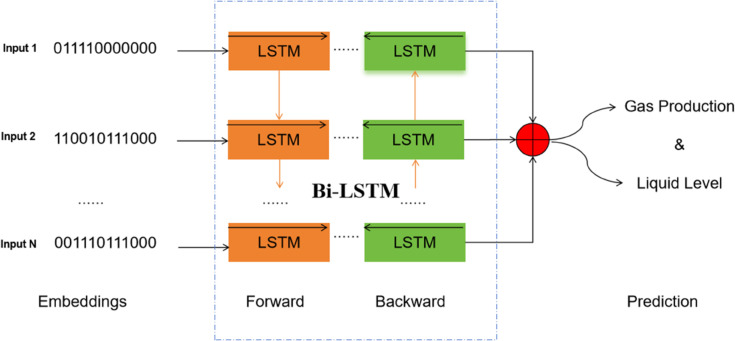
The introduced Bi-LSTM model used for gas production and liquid level predictions.

The Bi-LSTM (depicted in Fig 1) component is designed to process sequential data effectively. It takes in time-series data related to natural gas production and other relevant factors. The Bi-LSTM is capable of learning both forward and backward temporal dependencies in the data. This is crucial as it can capture complex patterns and relationships in the historical production data, which may have an impact on future production and the occurrence of liquid accumulation. For the natural gas production prediction task, the Bi-LSTM captures the historical production trends, potentially incorporating factors such as pressure, temperature, and flow rate data over a specific time period.

It consists of two sequential LSTM layers [34], which function in opposite directions to handle both forward and backward information states concurrently. Moreover, the Bi-LSTM architecture in question is made up of several essential constituents, namely an embedding layer, a dropout layer, a bidirectional LSTM layer, an attention layer, and an output layer. This model integrates multiple instances of attention mechanisms as described in the following:

### Model architecture and informer

The output of the Bi-LSTM is then fed into the Informer. The Informer is well-suited for handling long-sequence data and extracting important features. It employs a probabilistic attention mechanism, which significantly reduces computational complexity and memory requirements compared to traditional attention mechanisms. This allows the model to focus on the most relevant parts of the input data. In the context of this study, for the prediction of natural gas production, the Informer further refines the features extracted by the Bi-LSTM and generates predictions for the daily specific production. For the detection of liquid accumulation in wellheads, it classifies the situation into three categories: severe liquid accumulation, moderate liquid accumulation, and no liquid accumulation. The Informer’s ability to handle long sequences and its attention mechanism help in identifying key indicators and patterns that are associated with different levels of liquid accumulation.

**Fig 2:**
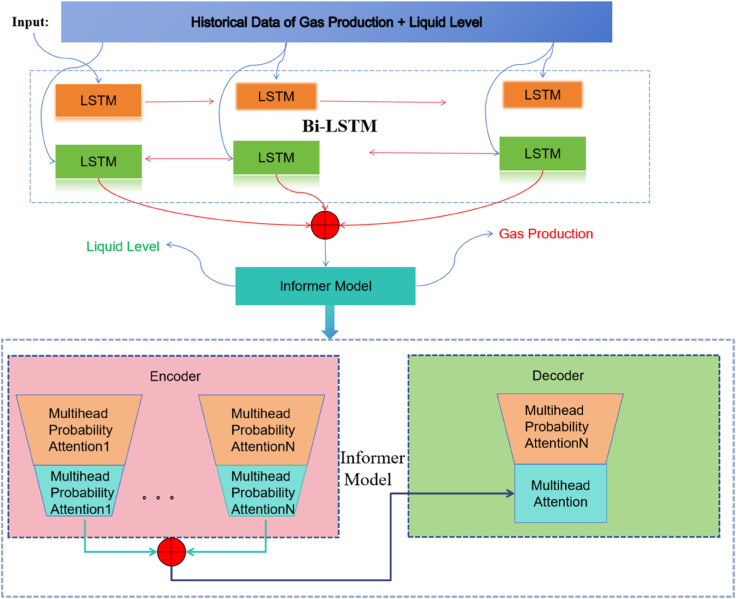
The architecture of the proposed model by integrating Bi-LSTM and Informer.

Meanwhile, the timestamp of each sample record is incorporated as supplementary information for the input of the proposed Informer-based pipeline (as depicted in Fig 2). It is worth noting that the internal structure of the Informer model can be obtained by referring to [36].

#### Probability attention mechanism.

**Fig 3:**
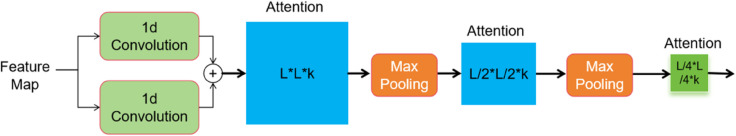
The proposed pipeline integrates a probabilistic attention approach. The parameter *L* represents the sequence length processed by the Conv1D layer, and *k* represents the number of feature maps generated within each attention module.

The Informer model utilizes a probabilistic attention mechanism, as illustrated in Fig 3. An attention module has the ability to concentrate on the crucial elements within the context that are relevant to the particular machine learning task in question.

The self-attention mechanism can be mathematically expressed as an input tuple, as demonstrated in Eq (1), which encompasses the query, key, and value components. This mechanism performs a scaled dot-product operation.

$$𝔸(Q,K,V)=softmax(\frac{QK^{T}}{\sqrt{d}})V,$$ (1)

where $Q\in {\mathbb{R}}^{L_{Q}\times d}$, $K\in {\mathbb{R}}^{L_{K}\times d}$, $V\in {\mathbb{R}}^{L_{V}\times d}$, and *d* represents the dimension of input.

The performance of the attention mechanism is frequently impeded by its quadratic computational complexity and high memory usage. To tackle these issues, the probability attention mechanism has been introduced, as described in Eq (2). This approach endeavors to lessen the computational load and memory demands related to traditional attention mechanisms, thereby enabling models to be more efficient while not compromising on performance.

$$𝔸(Q,K,V)=softmax(\frac{\hat{Q}K^{T}}{\sqrt{d}})V,$$ (2)

where the symbol $\hat{Q}$ represents a sparse matrix that has the same dimensions as matrix *Q*. Through the utilization of a multi-head architecture, the probability attention mechanism creates distinct sparse query-key pairs for each head, which is beneficial in avoiding significant loss of information.

#### Distilling operation.

The integration of the probability attention mechanism gives rise to a feature map that encompasses redundant combinations of the value *V*. To deal with this issue, the distillation process is employed to strengthen the prominent features within the feature map, as depicted in Fig 3. This operation has the ability to decrease the time dimension of the input data, and its mathematical formulation is presented as shown in Eq (3).

$$X_{j+1}^{t}=MaxPooling(ELU(Conv1D([X_{j}^{t}{]}_{A}))),$$ (3)

where $[X_{j}^{t}{]}_{A}$ represents the attention block, *Conv*1*D*(.) stands for a one-dimensional convolution operation with a kernel size of 5 along the temporal axis, and *ELU*(.) refers to the activation function in use. The max-pooling layer, which has a stride of 2, is able to halve the dimensions of *X*_*t*_, resulting in a significant reduction in memory consumption.

## Experiments

### Dataset collection and preprocessing

In this study, the production data samples were collected from multiple wellheads in Inner Mongolia, China over the past seven years. The data includes historical records of natural gas production on a daily basis, as well as measurements related to various parameters such as pressure, temperature, and flow rate. Additionally, data regarding the presence or absence of liquid accumulation in the wellheads was also gathered.

The collected data underwent several preprocessing steps. Firstly, missing values in the dataset were identified and handled. When the missing values were sporadic and the time-series nature of the data was to be preserved, interpolation techniques were considered. For a time-series *y*_*t*_ with missing values at time points *t*_*i*_, linear interpolation can be used. If $y_{t_{i-1}}$ and $y_{t_{i+1}}$ are the known values adjacent to the missing value $y_{t_{i}}$, the linearly interpolated value ${\hat{y}}_{t_{i}}$ is given by Eq (4):

$${\hat{y}}_{t_{i}}=y_{t_{i-1}}+\frac{\left(y_{t_{i+1}}-y_{t_{i-1}}\right)}{\left(t_{i+1}-t_{i-1}\right)}(t_{i}-t_{i-1}),$$ (4)

Secondly, the data was normalized to bring all the features to a comparable scale. This was achieved using standard normalization techniques such as min-max scaling. Min-max scaling transforms the data to a fixed range, typically [0,1]. For a feature *x*, the scaled value *x*_*scaled*_ is calculated as Eq (5):

$$x_{scaled}=\frac{x-x_{min}}{x_{max}-x_{min}}$$ (5)

where *x*_*min*_ and *x*_*max*_ are the minimum and maximum values of the feature *x* in the dataset.

The preprocessing stage is essential as it helps in improving the convergence speed and performance of the proposed model.

### Implementation details

The model was trained on the collected and preprocessed data. The dataset was divided into training, validation, and testing sets. The training set was used to optimize the model’s parameters. The loss function was carefully chosen based on the nature of the tasks. For the regression task of natural gas production prediction, a Root Mean Square Error (RMSE) loss function was used as it penalizes the model for large differences between the predicted and actual production values. For the classification task of liquid accumulation detection, a cross-entropy loss function was employed as it is suitable for multi-class classification problems. The validation set was used to monitor the model’s performance during training and to make decisions regarding early stopping or adjusting hyper-parameters.

The experiments were carried out using four NVIDIA RTX 3080 GPUs and the Pytorch framework [37]. In the proposed framework, the selected backbone models are LSTM and Informer, respectively. The input images were resized to achieve a uniform resolution in both width and height. Moreover, the pre-trained weight values obtained from the ImageNet dataset [38] were included as part of the initialization process for the proposed vision transformer. Additionally, a batch size of 32 was chosen, the Adam Optimizer was adopted as the hyper-parameter, a learning rate of 1e-9 was set, a depth of 8 was specified, a dropout rate of 0.1 was set, and the number of epochs was fixed at 200, as shown in Table 1.

**Table 1:** The hyper-parameter settings used in this study.

Parameter	Setting
batch size	32
optimizer	Adam (ϵ=1e−9)
depth	8
dropout rate	0.1
Learning rate	0.001 (Bi-LSTM) or 0.002 (Informer)
Epochs	200

The time-series dataset was split using a fixed-date approach to preserve temporal order: 80% (before June 29, 2023) for training, and 20% (June 29, 2023–2024) for testing. This split ensures the model is evaluated on data collected after the training period, mimicking real-world forecasting scenarios where future data is unseen during training.

### Evaluation metrics

The evaluation metrics employed in this study are RMSE and Mean Absolute Percentage Error (MAPE). The formula for RMSE is given by Eq (6):

$$RMSE=\sqrt{\frac{1}{n}\sum_{i=1}^{n}(y_{i}-{\hat{y}}_{i}{)}^{2}}.$$ (6)

where *n* represents the total number of samples or data points. *y*_*i*_ denotes the actual observed value for the *i*-th sample, and ${\hat{y}}_{i}$ is the corresponding predicted value.

The formula for MAPE is as Eq (7):

$$MAPE=\frac{1}{n}\sum_{i=1}^{n}\left|\frac{y_{i}-{\hat{y}}_{i}}{y_{i}}\right|\times 100\%$$ (7)

where *n* is the number of samples. The absolute difference between the actual and predicted values is divided by the actual value for each sample. These ratios are then summed up, divided by the number of samples, and finally multiplied by 100% to obtain the MAPE.

Accuracy is used as another evaluation metrics, which can be calculated using Eq (8):

$$Accuracy=\frac{TP+TN}{TP+TN+FP+FN}$$ (8)

where True Positives (TP) represent the number of samples that are correctly predicted as positive by the model. True Negatives (TN) refer to the number of samples that are correctly predicted as negative. False Positives (FP) are the number of samples that are wrongly predicted as positive when they are actually negative, and False Negatives (FN) are the number of samples that are wrongly predicted as negative while they are truly positive.

### Task definitions

To contextualize the model’s functionality, three distinct output tasks are explicitly defined, aligning with operational goals in natural gas production management:

- Point Forecasting: A task focused on predicting daily natural gas production to support resource allocation.
- Liquid Level Classification: A task involving the categorization of liquid accumulation into three states (severe, moderate, no accumulation) for maintenance prioritization.
- Prediction Interval Estimation (Extended Model): A task centered on generating 95% uncertainty intervals for production.

These tasks guide the model’s training and evaluation, ensuring alignment with practical operational needs.

### Experimental results

To evaluate the efficacy of the proposed model for natural gas production and liquid level detection forecasting, the state-of-the-art algorithms are used in the comparison experiments. These machine learning approaches include RF [39], DT [40], support vector machine (SVM) [41], CNN [42], RNN [43], and Bi-LSTM [31]. The comparative findings of the proposed models on the dataset can be seen in Tables 2 and 3.

**Table 2:** Comparison results between the state-of-the-art algorithms and the proposed deep learning models for natural gas production in terms of RMSE and MAPE.

Method	RMSE	MAPE
RF [39]	0.612	7.637
DT [40]	0.654	6.675
SVM [41]	0.702	4.741
CNN [42]	0.815	3.827
RNN [43]	0.821	3.849
Bi-LSTM [31]	0.451	3.664
Transformer [44]	0.412	2.173
Informer [32]	0.374	2.116
Autoformer [45]	0.339	2.005
Proposed approach	0.225	1.850

**Table 3:** Comparison results between the state-of-the-art algorithms and the proposed deep learning models for liquid level detection in terms of Accuracy.

Method	Accuracy
RF [39]	0.709
DT [40]	0.752
SVM [41]	0.776
CNN [42]	0.843
RNN [43]	0.857
Bi-LSTM [31]	0.879
Transformer [44]	0.913
Informer [32]	0.928
Autoformer [45]	0.951
Proposed approach	0.987

The comparison results presented in Tables 2 and 3 demonstrate the superiority of the proposed Informer model in both natural gas production forecasting and liquid level detection. For natural gas production, when evaluating the performance using RMSE and MAPE, the proposed Informer model achieved the lowest RMSE value of 0.225 and MAPE of 1.850. In contrast, traditional machine learning algorithms such as RF had an RMSE of 0.612 and MAPE of 7.637, while DT showed an RMSE of 0.654 and MAPE of 6.675. Among the neural network-based models, the Autoformer, which is already a relatively advanced model for time-series data, had an RMSE of 0.339 and MAPE of 2.005. The lower RMSE and MAPE values of the proposed model indicate that it can predict natural gas production more accurately, with smaller deviations from the actual values. This is crucial in the energy sector, as more accurate forecasts can lead to better resource management and operational planning. In the case of liquid level detection, measured by accuracy, the proposed Informer model also outperformed all the baseline models. It achieved an accuracy of 0.987, while the other models had accuracies ranging from 0.709 (RF) to 0.951 (Autoformer). The high accuracy of the Informer model implies that it can more effectively detect liquid levels, which is essential for ensuring the safe and efficient operation of related facilities.

Compared to the state-of-the-art models, the proposed model shows significant improvements in accuracy. In terms of efficiency, the Informer’s self-attention mechanism allows it to handle long-term dependencies with reduced computational complexity. This not only contributes to better accuracy but also implies that it can operate more efficiently than some traditional neural network models, especially when dealing with large-scale time-series data as in the natural gas production and liquid level detection datasets. Regarding computational cost, the reduced complexity of the Informer’s self-attention mechanism suggests that it may require fewer computational resources compared to more complex models that struggle with long-term dependencies.

**Fig 4:**
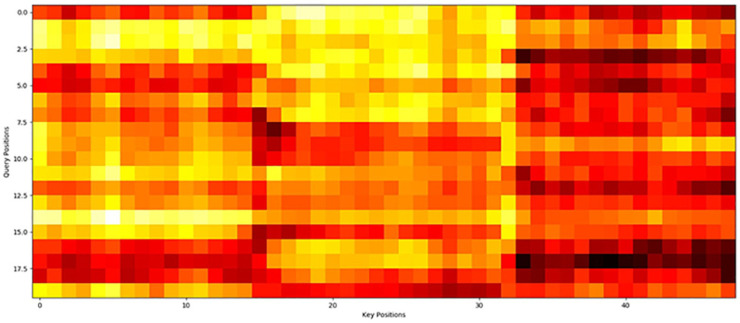
The attention map of the proposed approach on one batch of the manually collected dataset. Warmer colors denote higher attention weights, each entry in the attention map represents one time point the model deems for its prediction.

Moreover, to enhance the interpretability of the proposed model, the attention map of this model on the manually collected dataset was leveraged, as shown in Fig 4.

The attention map in Fig 4 illustrates the model’s focus on critical temporal segments and input features during processing. Warmer colors denote higher attention weights, indicating which time points the model deems most informative for its predictions. Notably, the model assigns lower weights to days with minimal variation in input variables, indicating it effectively filters non-informative segments. This pattern confirms that the model autonomously focuses on temporally relevant features known to impact production and liquid level dynamics, enhancing confidence in its decision-making logic.

**Fig 5:**
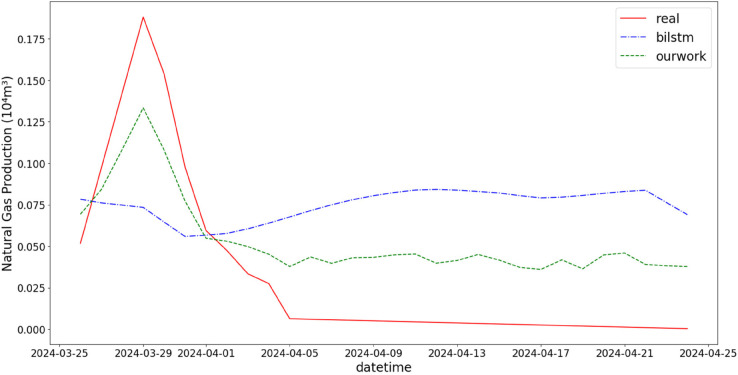
Comparison of predicted vs. actual natural gas production (March 25–April 25, 2024), demonstrating the model’s prediction accuracy during spring production peaks, which is valuable for seasonal resource allocation.

**Fig 6:**
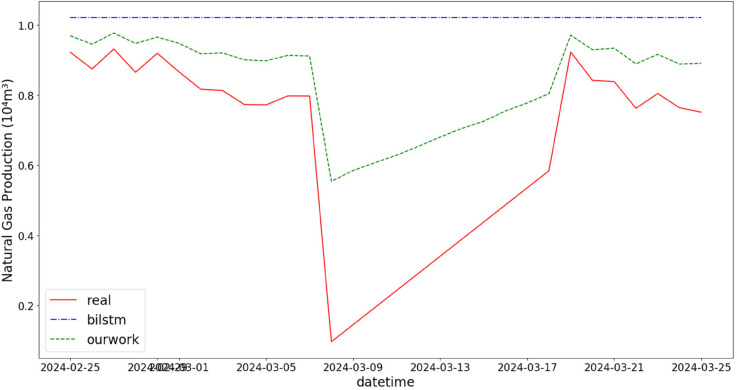
Comparison of predicted vs. actual natural gas production (February 25–March 25, 2024), showing the model’s effectiveness in capturing short-term fluctuations caused by operational adjustments, reflecting its adaptability to transient field conditions.

**Fig 7:**
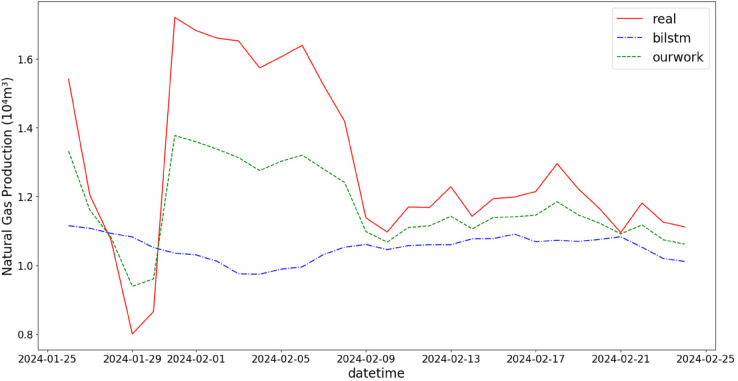
Comparison of predicted vs. actual natural gas production (January 25–February 25, 2024), with low deviation in low-temperature environments verifying the model’s stability under extreme climates.

**Fig 8:**
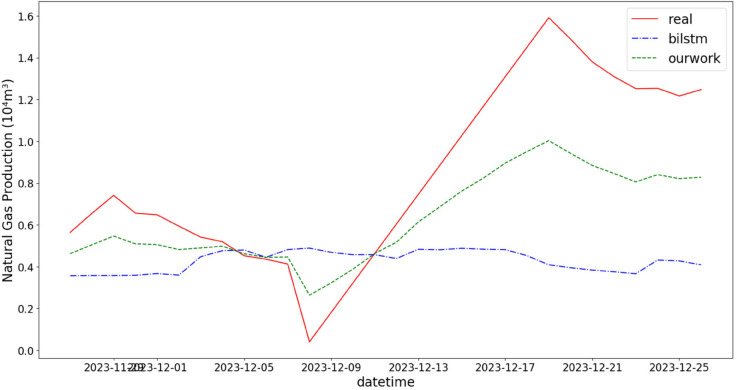
Comparison of predicted vs. actual natural gas production (December 29, 2023–January 25, 2024), illustrating the model’s ability to continuously predict cross-year production data, supporting long-term production planning.

**Fig 9:**
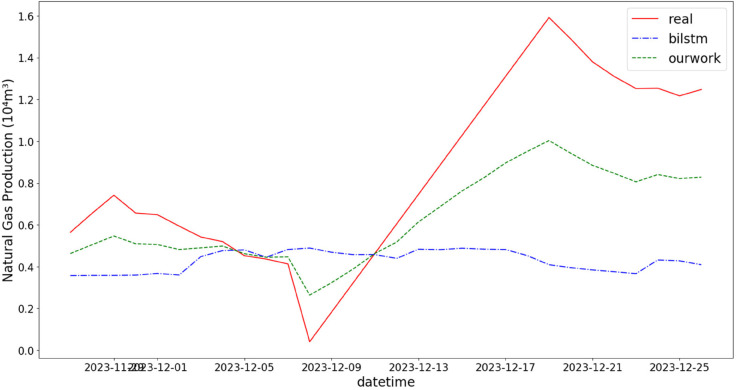
Comparison of predicted vs. actual natural gas production (November 29–December 25, 2023), accurately reflecting the production trend driven by increased winter heating demand.

**Fig 10:**
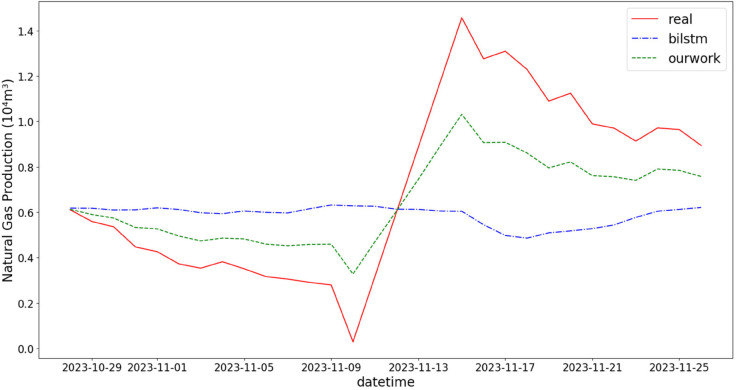
Comparison of predicted vs. actual natural gas production (October 29–November 25, 2023), with a low prediction error during the stable autumn production period.

**Fig 11:**
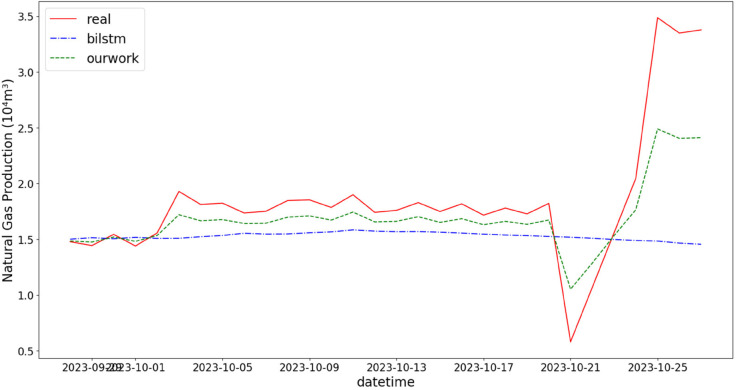
Comparison of predicted vs. actual natural gas production (September 29–October 25, 2023), demonstrating precise capture of minor production fluctuations, facilitating refined production control.

**Fig 12:**
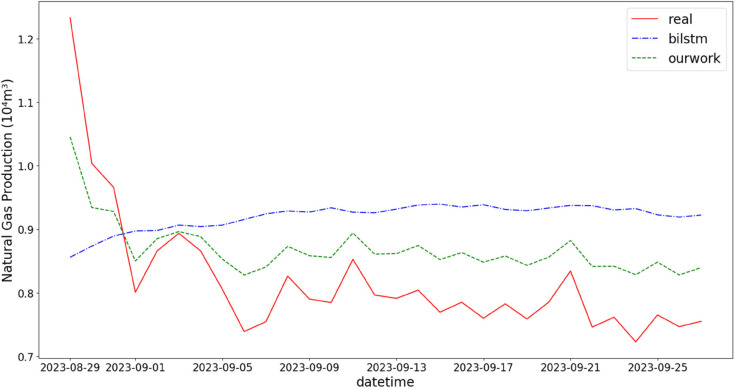
Comparison of predicted vs. actual natural gas production (August 29–September 25, 2023), with predictive performance under high-temperature conditions verifying the model’s environmental adaptability.

**Fig 13:**
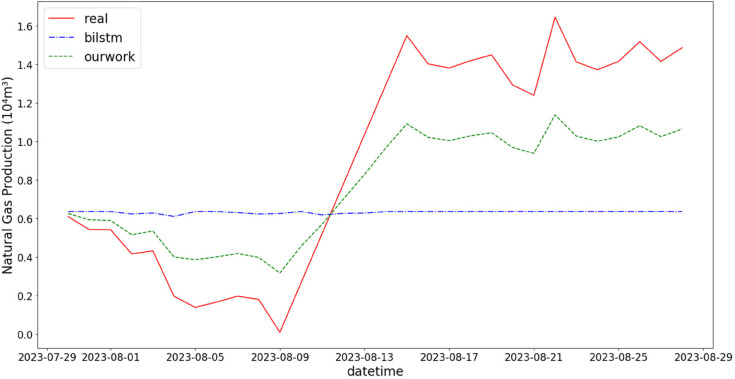
Comparison of predicted vs. actual natural gas production (July 29–August 29, 2023), maintaining consistency in long-sequence predictions to provide a basis for equipment maintenance planning.

**Fig 14:**
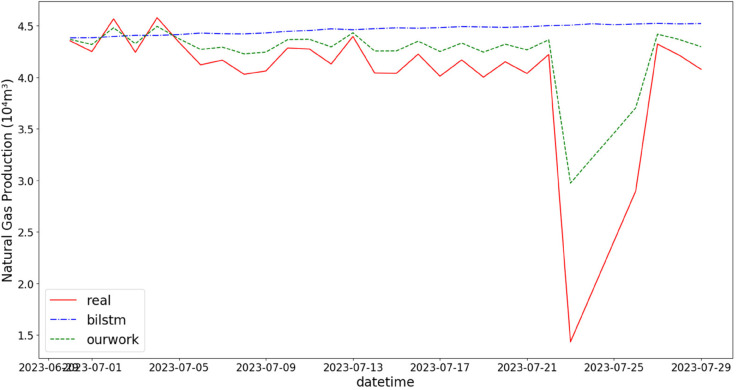
Comparison of predicted vs. actual natural gas production (June 29–July 29, 2023), with low deviation in the early testing phase laying the foundation for the model’s subsequent application reliability.

In addition, the comparison of natural gas production prediction between Bi-LSTM, Informer, and actual production data is provided, as shown in Figs 5, 6, 7, 8, 9, 10, 11, 12, 13, and 14, which focus on 2024 data to demonstrate the model’s performance on the most recent time period. Since the dataset spans 2017–2024, the models were evaluated on data before June 29, 2023 and reserved the remaining data as an independent test set to validate real-world predictive capability—this aligns with industry needs to forecast near-future production for resource allocation and maintenance scheduling. To note that the results shown from Figs 5 to 14 are derived from a single wellhead.

Overall, deep learning techniques in the literature have consistently outperformed shallow learning models when it comes to natural gas production and liquid level detection predictions. To note that shallow learning models refer to traditional machine learning approaches that lack deep hierarchical architectures. This demonstrates the benefits of using deep learning models to capture the internal structure of oil and gas-related applications. Nevertheless, shallow learning techniques might still hold significant worth in some applications.

### Ablation study

The ablation studies were firstly carried out to comprehensively validate the Bi-LSTM → Informer sequential pipeline. These ablation studies were designed to isolate and test the contributions of each component within different model architectures. These studies involved comparing different model configurations: using only Informer on the dataset, reversing the order of Bi-LSTM and Informer (Informer → Bi-LSTM), training a joint model of Bi-LSTM and Informer, and, for comparison, the original Bi-LSTM → Informer pipeline. The results of these experiments are presented in Table 4.

**Table 4:** Performance comparison of different model configurations in ablation studies.

Model Configuration	Production (RMSE)	Production (MAPE)	Liquid-Level (Accuracy)
Informer on Raw Data	0.453	4.278	0.924
Informer → Bi-LSTM	0.384	3.815	0.946
Bi-LSTM → Informer Pipeline	0.225	1.850	0.987

Table 4 shows the performance disparities among different model setups in the context of natural gas production prediction and liquid-level detection. When using only Informer on raw data, the model struggles to capture short-term temporal dependencies, as evidenced by the significantly higher RMSE and MAPE for natural gas production and the lower accuracy for liquid-level detection. This indicates that the initial feature extraction provided by Bi-LSTM is crucial for accurate predictions. In the reversed-order model (Informer → Bi-LSTM), although there is an improvement compared to using only Informer, it still falls short of the original pipeline. Bi-LSTM following Informer fails to optimally utilize the long-term dependencies captured by Informer for short-term prediction refinement. Overall, the results of these ablation studies strongly support the superiority of the Bi-LSTM → Informer sequential pipeline. It effectively combines the short-term feature extraction ability of Bi-LSTM with the long-term dependency handling of Informer, resulting in the best performance across both natural gas production prediction and liquid-level detection tasks.

To further explore the performance of the model in the face of noisy data, the experiments were conducted by adding Gaussian noise to the input data. Gaussian noise is a common form of data interference. In practical application scenarios, data may be affected by various random factors, resulting in similar noise. The Gaussian noise of different intensities was added to the training datasets of natural gas production and liquid level detection. Let the original data be x, and the new data x noisy after adding Gaussian noise is generated through Eq (9):

$$x_{noisy}=x+\epsilon \cdot \sigma \cdot N(0,1),$$ (9)

where *ε* is the noise intensity coefficient, *σ* is the standard deviation of the original data, and *N*(0,1) represents a standard normal distribution random variable. *ε* is set to 0.05, 0.1, 0.2, and 0.3, respectively.

For each noise intensity setting, the Bi-LSTM + Informer model was re-trained using the dataset with added noise and evaluated its performance on the test set. The evaluation metrics used are RMSE and MAPE for natural gas production prediction, and Accuracy for liquid level detection. The results of the re-trained model are provided in Tables 5 and 6.

**Table 5:** Performance of the model in natural gas production prediction after adding Gaussian noise.

Noise intensity coefficient *ε*	RMSE	MAPE
0 (no noise)	0.225	1.850
0.05	0.256	2.132
0.1	0.298	2.567
0.2	0.379	3.245
0.3	0.451	3.987

**Table 6:** Performance of the model in liquid level detection after adding Gaussian noise.

Noise intensity coefficient *ε*	Accuracy
0 (no noise)	0.987
0.05	0.972
0.1	0.951
0.2	0.923
0.3	0.897

As can be seen from Table 5, as the noise intensity coefficient *ε* increases, both the RMSE and MAPE indicators show an upward trend. When ϵ=0.05, the RMSE increases from 0.225 without noise to 0.256, and the MAPE increases from 1.850 to 2.132, indicating that the model performance begins to be affected to a certain extent, but still maintains relatively good prediction accuracy. When the noise intensity further increases to ϵ=0.3, the RMSE increases to 0.451 and the MAPE increases to 3.987, and the prediction error of the model increases significantly. This shows that noise has a significant negative impact on the performance of the natural gas production prediction model. As the noise intensity increases, the prediction accuracy of the model gradually decreases.

For the liquid level detection task, as can be seen from Table 6, as the noise intensity increases, the accuracy of the model gradually decreases. In the absence of noise, the accuracy is 0.987. When the noise intensity coefficient is 0.05, the accuracy drops to 0.972, and when *ε*=0.3, the accuracy drops to 0.897. This indicates that the model is also relatively sensitive to Gaussian noise in liquid level detection, and the presence of noise reduces the ability of the model to accurately detect the liquid level. Overall, through the experiment of adding Gaussian noise, it is found that the performance of the Bi-LSTM + Informer model will decline in the face of noisy data, but it can still maintain a certain degree of stability at a low noise intensity, providing a reference for the application of the model in the actual complex data environment.

To further validate the proposed model’s stability against noise compared to state-of-the-art methods, Gaussian noise analysis was extended to the top-performing models, including Transformer, Informer, and Autoformer. Using the same set of noise intensity coefficients (ϵ=0,0.05,0.1,0.2,0.3) applied to the input data, their performance was evaluated on natural gas production prediction and liquid level detection. The results, presented in Table 7, show that while all models exhibit performance degradation with increasing noise, the proposed model maintains the lowest error rates and highest accuracy across all noise intensities—particularly at higher noise levels (ϵ≥0.2), where its advantage becomes more pronounced. This confirms the proposed model’s superior robustness to real-world data perturbations.

**Table 7:** Gaussian noise analysis for top models within the state-of-the-arts and the proposed approach.

Model	*ε*	RMSE	MAPE	Accuracy
Transformer	0	0.412	2.173	0.913
	0.05	0.489	2.841	0.892
	0.1	0.567	3.529	0.865
	0.2	0.693	4.712	0.817
	0.3	0.825	5.984	0.763
Informer	0	0.374	2.116	0.928
	0.05	0.438	2.675	0.901
	0.1	0.512	3.218	0.876
	0.2	0.635	4.327	0.832
	0.3	0.759	5.413	0.789
Autoformer	0	0.339	2.005	0.951
	0.05	0.397	2.489	0.932
	0.1	0.462	2.973	0.905
	0.2	0.581	3.864	0.857
	0.3	0.698	4.921	0.812
Proposed Model	0	0.225	1.850	0.987
	0.05	0.256	2.132	0.972
	0.1	0.298	2.567	0.951
	0.2	0.379	3.245	0.923
	0.3	0.451	3.987	0.897

Moreover, to evaluate the influence of adding prediction intervals to the proposed model, the following ablation study was conducted. This is crucial as it helps us understand the contribution of this element to the overall effectiveness of the model. To note that the original Bi-LSTM + Informer model was designed to generate point predictions for natural gas production and liquid level. It consists of a Bi-LSTM layer that captures short and medium-term temporal dependencies and an Informer that deals with long-term dependencies. To introduce prediction intervals, the model was extended by applying quantile regression. Specifically, at the output layer of the Bi-LSTM + Informer model, multiple branches were added, each corresponding to a specific quantile. For a 95% prediction interval, 0.025 is taken for the lower bound, 0.975 is taken for the upper bound, and 0.5 is taken for the point prediction. During the training process, the loss function was adjusted to quantile loss. For a given quantile *τ*, the quantile loss $L_{\tau }(y,\hat{y})$ is calculated as Eq (10):

$$L_{\tau }(y,\hat{y})=\left\{ \begin{array}{cc} \tau \cdot |y-\hat{y}|, & \text{if }y\geq \hat{y}\\ (1-\tau )\cdot |y-\hat{y}|, & \text{if }y<\hat{y} \end{array}\right.$$ (10)

where *y* is the true value and $\hat{y}$ is the predicted value.

To comprehensively assess the impact of introducing Prediction Interval (PI), a set of evaluation metrics were leveraged. In addition to the traditional metrics for point predictions like RMSE and MAPE, the metrics related to prediction intervals were also incorporated, including Prediction Interval Coverage Probability (PICP) and Average Interval Width (AIW).

The experimental results for both natural gas production are presented in the Table 8:

**Table 8:** Performance comparison of original and extended models.

Model	Task	RMSE	MAPE	PICP (95% Interval)	AIW
Original Model	Production	0.225	1.850	-	-
Extended Model with PI	Production	0.348	1.964	0.947	0.600

As shown in Table 8, the PICP (95%) of 0.947 for the extended model indicates that 94.7% of actual values fall within the predicted intervals—closely aligning with the target 95% coverage. This near-perfect coverage confirms the model’s ability to quantify uncertainty effectively. For AIW, a value of 0.600 is considered acceptable in the context of natural gas production prediction, where typical daily production fluctuations range from 0.3 to 1.2 units in our dataset. A narrower interval would risk under-covering actual values, while a wider interval would reduce practical utility for operational decisions.

Finally, To identify the input variables most critical to prediction performance, we performed an ablation study by sequentially removing each feature (pressure, temperature, flow rate, historical liquid level records) from the input dataset and evaluating the resulting changes in model metrics. Table 9 summarizes the performance of the proposed model with each feature excluded, compared to the full model (all features included).

**Table 9:** Ablation study on feature importance for prediction performance.

Feature Removed	RMSE	RMSE Change (%)	Accuracy	Accuracy Change (%)
None (Full Model)	0.225	-	0.987	-
Wellhead Pressure	0.320	+ 42.2	0.935	–5.3
Casing Pressure	0.290	+ 28.9	0.955	–3.2
Production Hours	0.251	+ 11.6	0.974	–1.3

The results in Table 9 show that removing wellhead pressure data leads to the most significant degradation in performance: RMSE for production prediction increases by 42.2% (from 0.225 to 0.320), and accuracy for liquid level detection drops by 5.3% (from 0.987 to 0.935). This indicates wellhead pressure is the most influential variable. Casing pressure is the next critical feature, with its removal causing a 28.9% increase in RMSE and a 3.2% accuracy decline. Production hours records have relatively smaller impacts, though their exclusion still reduces performance, confirming their role in capturing secondary trends.

### Discussion

The results obtained from the proposed Bi-LSTM and Informer-based model for natural gas production prediction demonstrated clear effectiveness, with its performance closely tied to real operational dynamics. The low RMSE and MAPE values—consistently lower than baseline models across all time periods-reflect its ability to capture both gradual production trends and abrupt fluctuations. This aligns with the complementary strengths of the two components: Bi-LSTM’s bidirectional processing captured short-term pressure-driven fluctuations, while Informer’s attention mechanism preserved long-term seasonal patterns, ensuring the model balanced responsiveness and stability.

The Bi-LSTM and Informer sequential pipeline is theoretically supported by their complementary abilities, hierarchical feature extraction, and computational efficiency considerations. Bi-LSTM excels at capturing short-to-medium-term temporal dependencies and extracting fine-grained temporal features from the input sequence, while Informer is designed to handle long-term dependencies efficiently through its self-attention mechanism. By arranging them sequentially, Bi-LSTM first processes the data at a lower level to capture local temporal information, and then Informer takes these pre-processed features to capture long-term dependencies at a higher level, enabling hierarchical feature extraction. This not only makes full use of their complementary advantages but also reduces the complexity of data processing for Informer, thus improving computational efficiency. Additionally, compared to a joint model, the sequential pipeline has a more straightforward training process as the parameters of Bi-LSTM and Informer can be trained separately, which is easier to optimize and more likely to achieve better performance.

Regarding the detection of liquid accumulation in wellheads, the accuracy metrics provided valuable insights. The achieved accuracy values showed that the model was capable of distinguishing among the three categories (severe liquid accumulation, moderate liquid accumulation, and no liquid accumulation) with a reasonable degree of success. Overall, the model’s performance in this classification task suggested that the features extracted by the Bi-LSTM and further processed by the Informer were relevant for identifying the presence and severity of liquid accumulation, but there was still room for improvement in accurately capturing the subtler differences between the categories.

Overall, the results underscore the model’s dual value: its precision in tracking production trends supports proactive inventory management, while its liquid level detection reliability enhances safety. The deviations observed highlight actionable areas-such as incorporating environmental covariates—without undermining its core utility in operational decision-making.

When compared to existing methods in the literature for similar tasks of natural gas production prediction and wellhead liquid accumulation detection, the proposed model showed several advantages. Traditional statistical models often struggle to handle the complex time-series nature of production data and the multiple influencing factors. In contrast, the proposed approach, leveraging Bi-LSTM and Informer, was able to automatically learn these complex relationships. Some other machine learning models might have limitations in dealing with long sequences or might not be as efficient in extracting hierarchical features. The proposed model’s architecture overcame these issues to a large extent and achieved competitive performance in terms of prediction accuracy and classification metrics. However, it is important to note that some advanced deep learning models in other domains might have more sophisticated architectures or utilize larger datasets for training. While the proposed model performed well with the available data, further exploration of incorporating more extensive datasets or adopting more advanced architectures could potentially enhance its performance even more.

Despite the promising results, the proposed model has several limitations.

- The proposed model’s current generalization is constrained by the geological and operational uniqueness of the Inner Mongolia dataset, which includes specific reservoir types and extraction practices that may not align with other regions. For instance, shale gas reservoirs in Sichuan Basin exhibit higher clay content and require different fracturing techniques, which could introduce features not captured in our training data. Additionally, variations in data collection standards across regions may further reduce transferability. Without further validation, the applicability of the proposed model beyond Inner Mongolia is uncertain.
- In terms of real-time inference, the model requires substantial computational resources, specifically 4×RTX 3080 GPUs. The current hardware requirement stems from the model’s complexity, particularly the Informer’s multi-head attention mechanism and the large input sequence length. This poses challenges for edge deployment in field operations. Such environments often have limited computing power, energy resources, and storage capabilities, making it challenging to implement the proposed model without significant modifications. The lack of discussion on this aspect in the paper limits the practicality of the findings for real-time applications in the field.
- Finally, the proposed model is static, lacking a continual learning or adaptive mechanism to handle data drift in long-term deployment. Over time, changes in factors like production techniques, equipment degradation, or external environmental conditions can cause the data distribution to shift. Without an adaptive mechanism, the model’s performance may degrade over time, reducing its effectiveness for long-term prediction tasks.

## Conclusion

This study presented a novel prediction and detection model that combines Bi-LSTM and Informer for natural gas production and wellhead liquid accumulation analysis. The model architecture was designed to take advantage of the sequential learning capabilities of Bi-LSTM and the efficient feature extraction and long-sequence handling of Informer.

In the natural gas production prediction task, the evaluation using RMSE and MAPE metrics demonstrated that the model was able to capture the temporal trends and produce relatively accurate predictions. The combination of Bi-LSTM and Informer outperformed traditional methods such as RF and DT. Although it had its own characteristics compared to CNN and RNN, it showed competitive performance in handling the sequential nature of production data. For the wellhead liquid accumulation detection, the Accuracy metric indicated that the model could effectively classify the three categories of liquid accumulation. It had an edge over SVM in dealing with complex sequential wellhead data and improved upon Bi-LSTM alone with the addition of Informer’s mechanisms.

In the future, to improve cross-region applicability, the employment of transfer learning will be explored. First, the proposed model will be pre-trained on a diverse dataset from multiple regions, capturing general patterns in natural gas production and liquid level prediction. Then, it will be fine-tuned using the Inner Mongolia data. Validation will be done by testing the fine-tuned model on new basins or countries. This will broaden the model’s scope and help account for regional differences. For operational deployment, it is planned to create a lightweight model for edge and mobile use. Various techniques like pruning, quantization, and knowledge distillation will be used to compress the current model. Post-development, real-time inference tests on devices like Raspberry Pi will measure inference time and accuracy, assessing its feasibility in resource-constrained environments. This will make the predictive solutions more widely adoptable.
